# Impacts of climate change and mitigation policies on malt barley supplies and associated virtual water flows in the UK

**DOI:** 10.1038/s41598-019-57256-3

**Published:** 2020-01-15

**Authors:** D. O. Yawson, M. O. Adu, F. A. Armah

**Affiliations:** 1 Centre for Resource Management and Environmental Studies (CERMES), The University of the West Indies, Cave Hill Campus, P.O. Box 64, Bridgetown, BB11000 Barbados; 2 Department of Crop Science, University of Cape Coast, Cape Coast, Ghana; 3 Department of Environmental Science, University of Cape Coast, Cape Coast, Ghana

**Keywords:** Plant sciences, Environmental impact

## Abstract

Barley is a major ingredient for the malting industry which is highly sensitive and vulnerable to malt barley supply. The United Kingdom (UK) has the second highest malting capacity in the EU and the third largest malting industry in the world, supplying malt to major global breweries. Premium whisky, which has both economic and cultural significance for the UK, also makes sustainable malt barley supply critical for the UK. There is paucity of information on the sustainability of future supplies of malt barley in the UK, as much as it is in the world. This study applied a food balance approach to assess the combined effects of climate change and mitigation policies on UK malt barley balances for the 2030s, 2040 s, and 2050 s. Future yields of spring barley were simulated under the low, medium and high emissions scenarios (or LES, MES, and HES, respectively) for the three time slices. Future areas of land for barley production were obtained via land use change simulation in response to climate mitigation policies and aspirations of the UK. Future yields and land areas were combined to obtain total barley production, which served as a basis of supply. Per capita malt barley consumption was combined with future population to obtain demand. The gaps between demand and supply were then assessed. The results show large deficits in malt barley supplies for all combinations of climate change, land use and population, with adverse implications for the malting industry. Total malt barley supplies under current land area for barley and using the 90^th^ percentile yield, ranged from 1899 (LES, 2030s) to 2,437 thousand tonnes (HES, 2050s). The largest supply under climate mitigation land use scenarios ranged from 1,592 (LES, 2030s) to 2,120 thousand tonnes (HES, 2050s). Deficits in supply were observed for all climate mitigation land use scenarios and time slices, ranging from 128 to 585 thousand tonnes at 90^th^ percentile yield. However, surpluses were observed from the 2040s if current land area for barley remains unchanged. Imports to balance the observed deficits would result in large inflows of blue water to the UK, with adverse implications for global freshwater supply and environmental sustainability. It is concluded that even though spring barley yields in the UK could increase under projected climate change, reductions in croplands (due mainly to climate mitigation policies and aspirations) could combine with population growth to undermine the sustainability of malt barley supplies, both nationally and internationally.

## Introduction

Barley (*Hordeum vulgare* L.) is the fourth most important cereal crop in the world in terms of production^1^. Globally, malting (which underpins the malt-based beverage industry, as well as serve other food and beverage production) accounts for the second largest use of barley after feed use. Malt barley supply therefore underpins an economically and socio-culturally important industrial sector. Global consumption of malt-based beverages is projected to increase with incomes^2^. In countries where malt-based beverages have become an established part of culture, increased consumption can be anticipated regardless of health concerns^3^ and even without further increases in incomes. While climate change might adversely affect the supply and access to ‘luxury goods’ (such as alcoholic beverages) more than staple foods^4^, the impact of climate change on malting industry and its downstream industries have yet to attract appropriate level of research and policy attention. The malting industry is highly vulnerable to instability in supply of malt barley. To meet the demand for food, feed and industrial uses in 2050, global barley production will have to increase by about 54% over 2000 level^5^.

Barley is the second most important crop in the UK (after wheat) but the number one crop in Scotland. The United Kingdom (UK) is among the top ten producers in the world, with Russia being the largest producer. The leading producers (such as Russia, Ukraine, Australia) have larger harvested areas but yields are about twice lower than UK yields^1^. In the UK, about 60% of total barley grains produced is used for animal feed, 30% used for industrial purposes (mainly malting) while the remaining goes into minor uses such as seed, stock, food or waste^6^. In 2018, spring barley (the main source of malt barley) accounted for 59% of total UK barley production and total land area for barley was 1,157 thousand ha^7^. Malt barley production is important for the UK both economically and socio-culturally as it is a key ingredient in domestic brewing and distilling and the malt export sector. The UK has the second largest malt capacity in the EU, which accounts for 42% of global malting capacity and over 60% of world malt trade^8^. The UK malting industry is the third largest in the world and supplies malt to fourteen of the twenty largest brewers worldwide^9^. Particularly, the premium value and global brand of Scotch Whisky confers both economic and cultural significance on malt barley in the UK. Thus, the sustainability of the malting industry and its dependent value chains in the UK and other parts of the world is crucially contingent on adequate and stable supply of malt barley. Currently, a considerable proportion of UK malt barley is supplied from domestic production and overall barley import is low.

Climate change presents geographically varied risks to barley production. Due to the large proportion of barley used for animal feed, it is not surprising that the effect of future shocks to supply has been assessed mainly from food security or feed use perspective^10^. Globally, net barley production is projected to fall due to temperature and water stresses, with adverse implications for malting industry and its downstream industrial chains^4^. In the UK, it has been reported that spring barley production would remain viable, with potential increase in grain yield under projected climate change^11^. However, the question remains whether this yield gain could be sufficient for stable supply of malt barley. Given the projected gains in yield, total production or supply would depend mainly on total land area allocated to barley in the future, while population and markets would drive demand. Studies have shown that there is potential for reductions in UK croplands in the future, due mainly to climate change policies^12–14^, or agricultural policies and market signals^10,15^. It has been shown that land use change effects can offset the potential gains in yield to dampen total production, and together with population growth, lead to overall deficits in feed barley supply^10^.

In spite of malt barley’s economic and cultural significance to the UK, and the sensitivity of the malting industry to stability in supply, there is paucity of information on the future balances of malt barley under projected environmental change in the UK. In addition, due to the impacts of climate, land use and population change on future balances of malt barley in the UK, it is important to assess the direction of flow of virtual water (water embedded in a crop commodity that is traded^16^) due to deficits (import) or surplus (export). This would help assess potential UK contribution to global freshwater savings or losses. This paper adopted a food balance approach to assess future balances of UK malt barley and potential virtual water flows associated with import or export in response to deficits or surpluses under projected climate, land use and population change.

## Methods

### Food balance approach

The approach used in the current study was based on the balances or distribution of food supplies and utilization as captured in the food balance sheet (FBS) from the Food and Agriculture Organization (FAO) of the United Nations. The FBS is one of the major sources of data for analysing patterns of food supply, utilization, and balances at country or international level and over long time period. Other sources of such data would include household budget surveys and individual dietary surveys^17^, which are expensive to collect, often unavailable and difficult to access. The FBS has high utility for national and international studies as the datasets are consistent, comparable, and temporally continuous. In addition, the easy and free accessibility of the FBS data makes it the most widely used in national and international studies on food balances or security. The FBS of a country shows a 3-year average supply and uses of food items in the given country for the given reference year^18^. The FBS divides food items into supply and utilization. Total supply of a given food item is the sum of the total domestic production and imports, adjusted for changes in stocks occurring since the beginning of the reference period. Regarding utilization, the total supply of food items is distributed according to quantities exported, used for feed and seed, processed for food and non-food uses, postharvest losses, and the proportion available for food use or direct human consumption^18^. The per capita supply of a given food is obtained as the quotient between the proportion available for human consumption and the total population. The FBS can therefore be useful for estimating food shortages or surpluses, projecting future food requirements and analysing policy implications of food production and trade^17–19^. The current study used the supply and utilization in the FBS as a basis for analysing future deficits or surplus in malt barley supply and associated virtual water flows in the UK.

For the baseline, information on barley supply and utilization in the FBS of 2009 for the UK was used. From this FBS, malt barley accounted for 1.7 million tonnes out of the 5 million tonnes of total barley supplied for domestic uses. Feed use accounted for about 60% of total barley supplied for domestic uses. Total imports and exports were 115 and 633 thousand tonnes respectively. It is noteworthy that imported barley in the UK largely goes into malting. Per capita malt barley was 28 kg/yr. To obtain future distribution of barley on the utilization side, the current proportionate (or percentage) distributions were calculated and assumed to remain unchanged under the future time slices. Thus, total quantity of barley supplied for domestic uses was 83% of total production. Total malt barley was 28.7% or 34.6% of total barley produced or supplied for domestic uses, respectively. These proportions and per capita malt barley supply were used to calculate future supplies (from production) and demand (in conjunction with projected population data). Details of this approach can be found in Yawson^19^.

#### Future malt barley supply

The supply side of the FBS is based mainly on domestic production and imports. Future barley grain yields in the UK were simulated under three climate change emissions scenarios (low, medium, and high; or LES, MES, and HES, respectively) for three time slices (2030s, 2040s, and 2050s) and for the 14 UK administrative regions. The FAO AquaCrop model^20,21^ was used for the crop simulations. Projected climate data were obtained from the publicly accessible UK Climate Projections 2009 (UKCP09) database (see Murphy *et al*.^22^ for detailed description). The UKCP (from the UK Met Office Hadley Centre Climate Programme) provides up to date data on projected climate change for the UK over the course of the 21^st^ century, using ensembles of the Met Office Hadley Centre climate model and other global climate models^22^. The UKCP09 provides projected data for climate variables, averaged over seven overlapping 30-year time periods, for each of three IPCC’s Special Report on Emission Scenarios (SRES) scenarios, namely, the A1FI (High Emission Scenario or HES), A1B (Medium Emission Scenario or MES), and B1 (Low Emission Scenario or LES)^22^. Each SRES scenario represents a different narrative of and assumptions on socio-economic development pathway and associated greenhouse gas emissions. The A1FI represents a development pathway based on intensive use of fossil fuels. The B1 represents the use of efficient and clean technology, and less intensive use of materials. The ranges and uncertainties of projected temperature and precipitation for the three emission scenarios have been reported^19,22^. The Weather Generator (version 2) embedded in the UKCP09 (see Jones *et al*.^23^) was used to generate future daily climate variables for the three emissions scenarios, time slices, and the 14 UK administrative regions. The resulting files were processed in the AquaCrop-compatible format for the simulations.

Soil hydraulic data required by AquaCrop for the simulations were obtained from the new Soil Information System used in the Monitoring Agriculture with Remote Sensing (MARS) Crop Yield Forecasting System (MCYFS) for the EU^24^. In this database, a soil mapping unit (SMU) comprises several Soil Typological Units (STUs) with attributes describing the properties of the soils. This dataset was imported in ArcGIS 9.1 (ESRI™, USA), processed and the area covering the UK was clipped. Attribute tables were then processed appropriately and joined to obtain a single database on UK. Soil attributes or properties such as soil type, saturated water content, water content at permanent wilting point, water content at field capacity, and total available soil water were extracted and formatted in AquaCrop-compatible format. Subsequently, drainage characteristics such as drainage coefficient (tau), saturated hydraulic conductivity and curve number for surface runoff were generated in AquaCrop using the input soil data from the SINFO dataset as described earlier. Soil fertility was considered as optimal (no fertility stress) and no other management strategies were considered.

The future simulation was based on the barley genotype Westminster, which was on the HGCA (Home-Grown Cereals Authority) Recommended List, widely grown in the UK both as winter and spring barley crop, for feed and malt, and high-yielding. The genotype Westminster was part of a calibration and validation study using AquaCrop^19^. The data (crop, soil, weather) for the calibration and validation were obtained from field experiments at the James Hutton Institute (Dundee, UK). The root mean square error (RMSE) of calibration was 8.1%. The yield differences for the two years of validation were 0.91 and 1.73 tons ha^−1^ (see^19^ for details). The simulations for future yields were done for spring barley, under rain-fed conditions. The model parameters were based mainly on the calibration and validation information presented earlier, information from Raes *et al*.^20^, and personal communications with scientists at The James Hutton Institute and based on thermal time. The search for an appropriate sowing date for each UK region was restricted to the range of optimum sowing period (±1 week) recommended by the HGCA, outside of which could result in yield penalties. To this end, the AquaCrop model setup was forced to the 1990 regional baseline yields by changing only the sowing date until the simulated yield approximated the observed yield. The first date that gave the closest match between the simulated and observed yields was selected as the sowing date for that particular region. The reliability of the sowing dates and the model setup were assessed by comparing the observed and simulated yields for the baseline period 1980–1989. Based on the regional averages, the prediction error for the simulated and observed yields for the period 1980–1989 and for the UK was 0.35 tons ha^−1^. Apart from the search for sowing dates, no changes were made in the model setup and no variations in field or fertility management were done. For all the simulations, no field management was specified, there was no fertility stress, and initial soil water content was set to field capacity. The simulations were run as multiple run projects using the AquaCrop plug-in program version 3.1 + . The barley grain yield for the regions were averaged to obtain the corresponding information for the UK^19^.

Future cropland areas in the UK for the three time slices were obtained from Annex 2 of the study on greenhouse gas emissions by sources and removals by sinks due to land use, land use change and forestry in response to prevalent climate policies and mitigation policy aspirations and priorities of the UK^13^. For this study, five land use scenarios were developed^13^, comprising two baseline scenarios (BL), Low scenario (Low), the Central scenario (Central), and the Stretch scenario (Stretch). For detailed descriptions of these scenarios, see Thomson *et al*.^13^. The central and the stretch scenarios are middle of the road and the most ambitious, respectively. Because the total cropland area under the two baseline scenarios were the same, they were represented as one in the current study.

To obtain future area of land under barley production from the cropland areas in Thomson *et al*.^13^, the average proportion or percentage of barley land area in total UK cropland area for the period 2000–2012 was calculated. For this period, the average barley land area was 1,026 thousand ha or 16.36% of total cropland area. Hence, 16.36% of the cropland areas obtained from Thomson *et al*.^13^ was calculated to represent proportionate barley land area for the three time slices under the respective land use scenarios. In addition, the average land area of barley for the period 2000–2012 (i.e. 1,026 thousand ha) was used as a business-as-usual (BAU) scenario to capture what the situation would be should the current land area remain unchanged. Future total barley production for the time slices and emissions scenarios were obtained as the product of respective yields (90^th^ and 50^th^ percentiles) and total land area under barley cultivation. Total malt barley supply was then derived using the representative proportion as presented earlier.

#### Future Malt Barley demand, balances and virtual water flows

In the current study, future total malt barley demand was obtained as the product of per capita malt barley supply (derived from the FBS) and projected UK population. That is, the current per capita supply or use was assumed to remain unchanged to the future. Projected UK population for the three time slices were obtained from the UK National Population Projections from the UK Office of National Statistics. These population projections are based on four scenarios: the low, high and constant fertility and balanced long-term migration. The future UK population used in the current study was based on the constant fertility scenario.

### Virtual water flows

Future surplus or deficits will determine the direction of net virtual water flow in correspondence to export or import, respectively. The virtual water content (VWC) of UK barley was estimated as

$${\rm{VWC}}\,({{\rm{m}}}^{3}\,{{\rm{ton}}}^{-1})=10\ast (\frac{ETc}{Yield})$$

With ETc denoting crop evapotranspiration (mm) and the 10 is a scalar to ensure consistent units^25^. The magnitudes of ETc and the 90^th^ and 50^th^ percentile grain yields of UK barley were obtained from the simulations.

Total virtual water (TVW) associated with malt barley, import or export (whether green or blue water) was obtained as:

$${{\rm{TVW}}}_{{\rm{e}},{\rm{i}}}({{\rm{m}}}^{3})=VWC\ast T$$

where T denotes total quantity (tonnes) of malt barley under consideration.

Net virtual water flow (NVW) was estimated as:

$${\rm{NVW}}({{\rm{m}}}^{3})=TVWi-TVWe$$

where *e* and *i* denote export and import, respectively. A positive NVW value implies net virtual water flow to the UK, and vice versa. Where NVW was negative, it was used to indicate total volume of water that the UK could potentially export. A sum of the blue and green NVW provides the total NVW. It was assumed that import will only be in the order of magnitude of deficits while export will correspond to the magnitude of surplus.

Source and sink of virtual water flows were considered in terms of UK’s current trade partners with regard to barley. The trade information (quantities and partners) were retrieved from the FAOSTAT trade database for the baseline period. Out of 21 main trading partners for barley, the top eight partners accounted for about 95% of total UK barley imports. These were Ireland, France, Germany, Ukraine, Spain, Denmark, Sweden, and Italy. The remaining countries contributed less than 2% each and so were aggregated as the rest of the world. Assuming that these countries would remain the UK’s main partners with regard to barley trade in the future, the VWC of barley from these countries were retrieved. The average VWC of barley from these countries were used to calculate average virtual water flows to the UK due to malt barley import. The VWC of barley for the partner countries were obtained from the WaterStat Database of the Water Footprint Network^26^. The green and blue virtual water flows were calculated separately and then aggregated to obtain total virtual water flows.

## Results

### Future malt barley supply

The yields did not vary much, with the largest standard deviation for all emission scenarios and time slices being 1.08. Total barley production was based on the 90^th^, 50^th^ and 10^th^ percentile yields in combination with the land area under the different scenarios for each of the time slices and emissions scenarios (Table 1). The 10^th^ percentile yields did not vary substantially from the 50^th^ percentile yields. Obviously total production at 90^th^ percentile yield is largest for all land use scenarios, emissions scenarios and time slices. Again, because yields increased from the LES to the HES and from the 2030s to the 2050s, the magnitudes of total production for all land use scenarios followed a similar trend. Based on the 10^th^ percentile yields, the largest and smallest total production was 7521 and 4326 thousand tonnes (BL, MES, 2030). For all the scenarios, the magnitudes of total production under the current area of land for barley (BAU) were considerably larger than the values for all other land use scenarios derived in response to climate mitigation (Table 1). For the BAU, and at 90^th^ percentile yield, total production ranged from 6,614 (LES, 2030s) to 8,486 thousand tonnes under the HES in the 2050s. For the remaining land use scenarios and at 90^th^ percentile yield, total production was largest under the Stretch scenario and smallest under the BL scenario for all emissions scenarios and time slices. However, the differences between the total production under the climate mitigation land use scenarios were not as large as the difference between the BAU and these other land use scenarios. For the Central (middle of the road) scenario, the smallest and largest total production were 5,491 (LES, 2030s) and 7,315 thousand tonnes under the HES in the 2050s and were the same as for the Low scenario due to same magnitude of land area. The difference between total production under the HES and MES widens from the 2040s.

**Table 1:** Projected total UK barley production (‘000 tonnes) under the land use scenarios, based on the 90^th^, 50^th^ and 10^th^ percentile yields.

	Emission scenario	Yield percentile	BAU	BL	Low	Central	Stretch
2030	LES	90^th^	6614	5415	5491	5491	5543
		50^th^	6168	5049	5120	5120	5169
		10^th^	5858	4796	4863	4863	4910
	MES	90^th^	7585	6209	6296	6296	6357
		50^th^	6918	5663	5743	5743	5798
		10^th^	5284	4326	4386	4386	4428
	HES	90^th^	7603	6224	6311	6311	6372
		50^th^	6752	5528	5605	5605	5659
		10^th^	5941	4863	4931	4931	4979
2040	LES	90^th^	6749	5584	5710	5710	5763
		50^th^	6370	5270	5389	5389	5440
		10^th^	6074	5026	5139	5139	5187
	MES	90^th^	7874	6515	6662	6662	6725
		50^th^	7189	5948	6082	6082	6139
		10^th^	5387	4457	4557	4557	4600
	HES	90^th^	8082	6687	6838	6838	6902
		50^th^	7315	6053	6189	6189	6247
		10^th^	6607	5467	5590	5590	5643
2050	LES	90^th^	6928	5793	5972	5972	6027
		50^th^	6627	5542	5713	5713	5766
		10^th^	6259	5233	5395	5395	5445
	MES	90^th^	8216	6870	7082	7082	7148
		50^th^	7686	6427	6625	6625	6686
		10^th^	6361	5319	5483	5483	5534
	HES	90^th^	8486	7096	7315	7315	7383
		50^th^	8080	6756	6965	6965	7029
		10^th^	7521	6289	6483	6483	6543

In the 2030s and under the BAU, the total production decreased by 6.76%, 8.78% and 11.19%, respectively for the LES, MES and HES at the 50^th^ percentile yields. The corresponding decreases were 5.62%, 8.70 and 9.49%, respectively for the 2040s while the decreases for the 2050s were 4.34%, 6.45% and 4.78%, respectively. Approximately, similar rates of reductions would be observed for the remaining land use scenarios as the yield values were constant for all the land use scenarios. These percentage reductions would remain unchanged for the subsequent malt barley supply from total production which are based on the 90^th^ and 50^th^ percentile yields henceforth.

Because the barley supply for domestic use (Table 2) is a constant proportion of total production for all land use, emissions scenarios and time slices, the patterns and percentage reductions between the 90^th^ and 50^th^ percentile yields observed for total production (Table 1) are repeated. Total barley supplied for domestic use (from total production) under the BAU and at 90^th^ percentile yield ranged from 5,490 (LES, 2030s) to 7,044 thousand tonnes (HES, 2050s) (Table 2). Similarly, total barley supply for domestic uses under the Central scenario ranged from 4,557 (LES, 2030s) to 6,072 thousand tonnes (HES, 2050s), and the corresponding values for the Stretch were 4,601 and 6,128 thousand tonnes, respectively.

**Table 2:** Projected total barley supply for domestic uses from total production (‘000 tonnes).

	Emission scenario	Yield percentile	BAU	BL	Low	Central	Stretch
2030	LES	90^th^	5490	4494	4557	4557	4601
		50^th^	5119	4191	4250	4250	4291
	MES	90^th^	6295	5154	5226	5226	5276
		50^th^	5742	4701	4766	4766	4812
	HES	90^th^	6310	5166	5238	5238	5289
		50^th^	5604	4588	4652	4652	4697
2040	LES	90^th^	5601	4635	4739	4739	4784
		50^th^	5287	4374	4473	4473	4515
	MES	90^th^	6536	5408	5529	5529	5582
		50^th^	5967	4937	5048	5048	5096
	HES	90^th^	6708	5550	5675	5675	5729
		50^th^	6072	5024	5137	5137	5185
2050	LES	90^th^	5750	4808	4957	4957	5003
		50^th^	5501	4600	4742	4742	4785
	MES	90^th^	6819	5702	5878	5878	5933
		50^th^	6379	5334	5499	5499	5550
	HES	90^th^	7044	5890	6072	6072	6128
		50^th^	6706	5608	5781	5781	5834

Based on the proportionate distribution for utilization, according to the food balance approach, total malt barley supply from domestic production (using the 90^th^ percentile yield) under the BAU ranged from 1,899 to 2,437 thousand tonnes under the LES in the 2030s and HES in the 2050s, respectively (Table 3). As observed earlier, malt barley supply under the BAU are larger than the supply under the remaining land use scenarios. Thus, future malt barley supply based on the projected crop areas would be lower than the supply if current land area for barley is maintained to the future. Apart from the BAU, the Stretch scenario has the largest malt barley supply, ranging from 1,592 (LES, 2030s) to 2,120 thousand tonnes (HES, 2050s) while the supply under the BL scenario ranged from 1,555 (LES, 2030s) to 2,038 (HES, 2050s). It is noteworthy that the largest malt barley supply of 2,120 thousand tonnes under the climate mitigation land use scenarios (Stretch, HES, 2050s) is approximated by total supply under the MES in the 2030s for the BAU, indicating significant reduction in supply capacity.

**Table 3:** Projected malt barley supply (‘000 tonnes) at 90^th^ and 50^th^ percentile yields for the different land use scenarios, emissions scenarios and time slices.

	Emission scenario	Yield percentile	BAU	BL	Low	Central	Stretch
2030	LES	90^th^	1899	1555	1577	1577	1592
		50^th^	1771	1450	1470	1470	1485
	MES	90^th^	2178	1783	1808	1808	1826
		50^th^	1987	1626	1649	1649	1665
	HES	90^th^	2183	1787	1812	1812	1830
		50^th^	1939	1587	1610	1610	1625
2040	LES	90^th^	1938	1604	1640	1640	1655
		50^th^	1829	1514	1548	1548	1562
	MES	90^th^	2261	1871	1913	1913	1931
		50^th^	2064	1708	1747	1747	1763
	HES	90^th^	2321	1920	1964	1964	1982
		50^th^	2101	1738	1777	1777	1794
2050	LES	90^th^	1990	1664	1715	1715	1731
		50^th^	1903	1591	1641	1641	1656
	MES	90^th^	2359	1973	2034	2034	2053
		50^th^	2207	1846	1903	1903	1920
	HES	90^th^	2437	2038	2101	2101	2120
		50^th^	2320	1940	2000	2000	2019

### Future malt barley demand and balances

**Figure 1:**
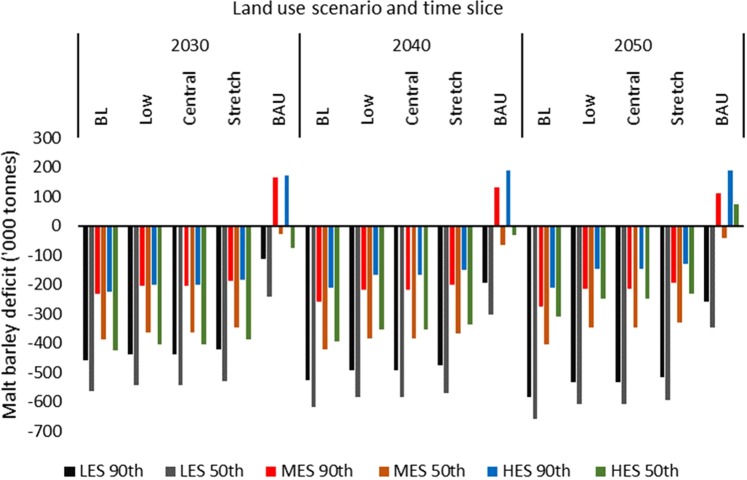
Projected deficits in malt barley supply (‘000 tonnes) based on the Constant fertility scenario.

In the 2030s, projected UK population was lowest under the Low fertility scenario but the Balanced Long-Term Migration scenario showed the lowest in the 2040s and 2050s (Table 4). The total demand for malt barley varied accordingly, ranging from 1,946 thousand tonnes (2030s) to 2,302 thousand tonnes (2050s). Subsequently, the malt barley balances (difference between demand and supply) were based on the Constant fertility scenario. Deficits were observed for all combinations of land use and emissions scenarios and for all time slices except for the BAU under the MES/HES-2030s-90^th^ percentile yield, MES- and HES-2040s/2050s-90^th^ percentile yield, and HES-2050s-50^th^ percentile yield (Fig. 1). Thus, if even current land area is maintained to the future, there could be surplus over demand at 90^th^ percentile yields under the MES and HES right from the 2030s. As expected, the deficits in malt barley supply were lowest under the BAU and largest under the BL (but comparatively larger for all the climate mitigation land use scenarios). The deficits under the BAU and at 90^th^ percentile yield ranged from 114 thousand tonnes (LES, 2030s) to 259 thousand tonnes (LES, 2050s) due to the relatively lower yields under the LES. The deficits under the Stretch scenario ranged from 128 thousand tonnes (HES, 2050s) to 517 thousand tonnes (LES, 2050s). The interactive effect of population, yield and land use can be deduced from the patterns across the time slices. For example, under the BAU, while the deficits under the LES increased from the 2030s to the 2050s, the surplus under the MES decreased across the time slices but increased for the HES. An increase in deficits across the time slices were observed for the LES and MES under the Stretch scenario while the values for the HES decreased due to higher yields countering the effect of population or demand.

**Table 4:** Future population and corresponding malt barley demand.

Scenario	Population (million)			Malt Barley Demand (‘000 tonnes)		
	2030	2040	2050	2030	2040	2050
High	72.8	77.3	82.2	2038.4	2164.4	2301.6
Low	69.5	72	74	1946	2016	2072
Constant	71.9	76.1	80.3	2013.2	2130.8	2248.4
Balanced Long-Term Migration	70.3	71.5	71.9	1968.4	2002	2013.2

### Virtual water flows

**Figure 2:**
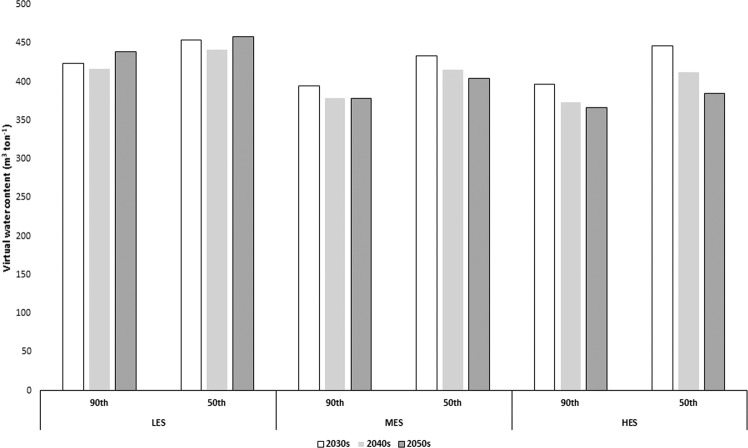
Virtual water content of UK spring barley grain at 50^th^ and 90^th^ percentile yields under low, medium and high emissions scenarios (LES, MES, and HES, respectively) in the 2030s, 2040s, and 2050s.

The virtual water content (VWC, m^3^ ton^−1^) of UK spring barley decreased from the LES to the HES and from the 2030s to the 2050s for the MES and HES (Fig. 2), suggesting gains in water productivity. As can be expected, the VWC values at 90^th^ percentile yields were lower than for the 50^th^ percentile yields. At 90^th^ percentile yield, the VWC ranged from 366 (HES, 2050s) to 438 (LES, 2050s). The observed VWC of UK spring barley was 100% green water.

**Figure 3:**
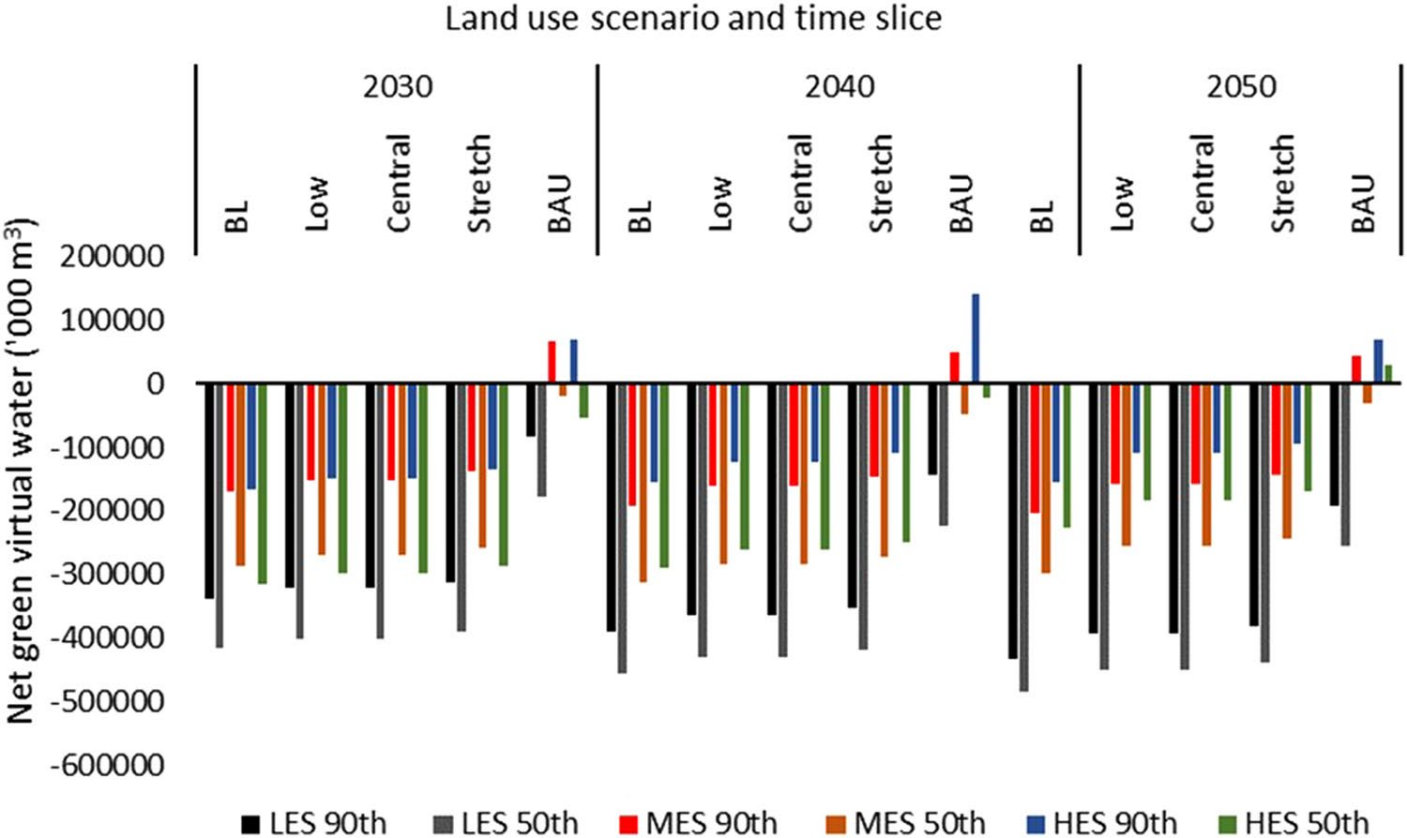
Net green virtual water flows (‘000 m^3^) based on malt barley balances.

**Figure 4:**
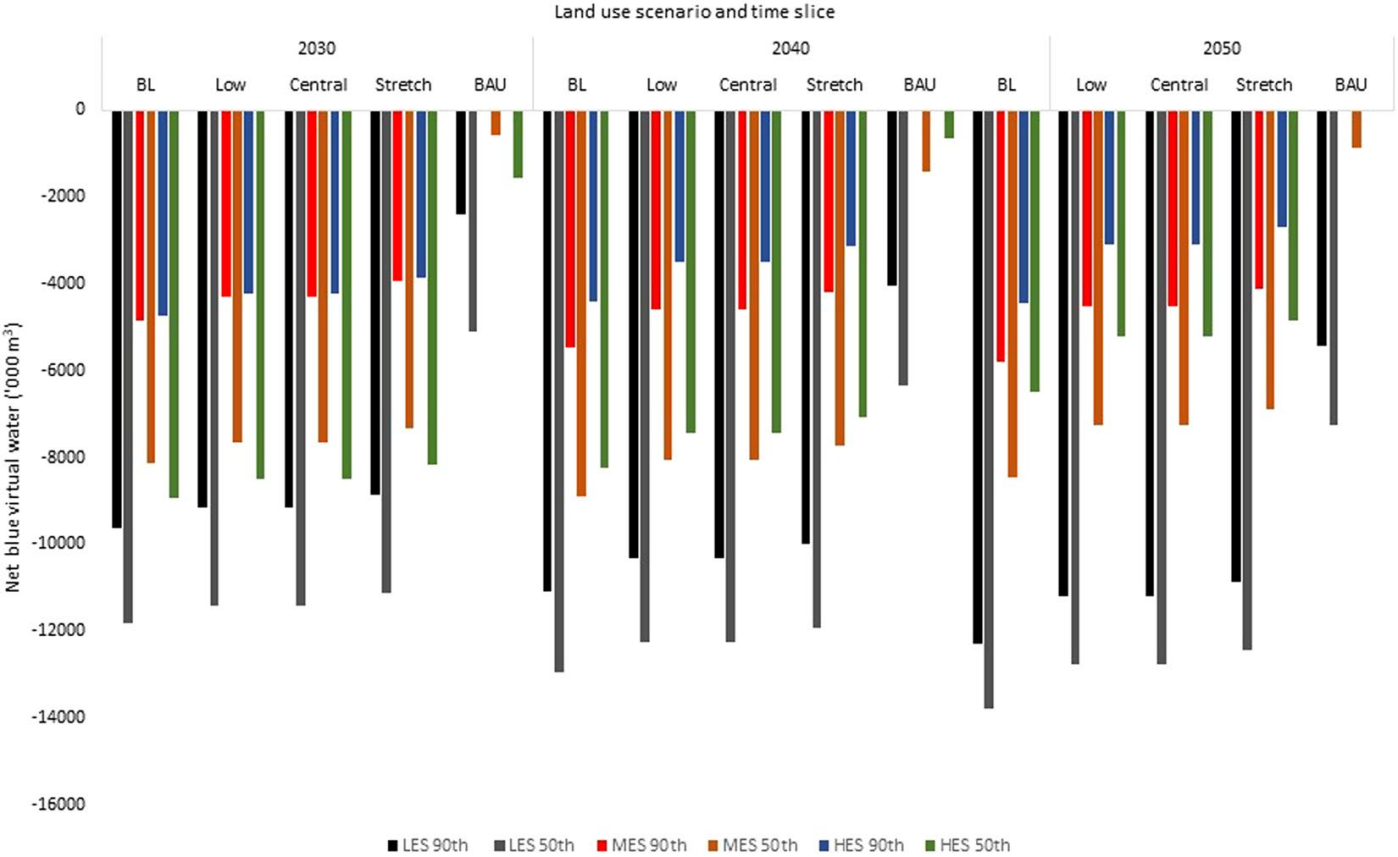
Net blue virtual water flows (‘000 m^3^) based on malt barley balances.

**Figure 5:**
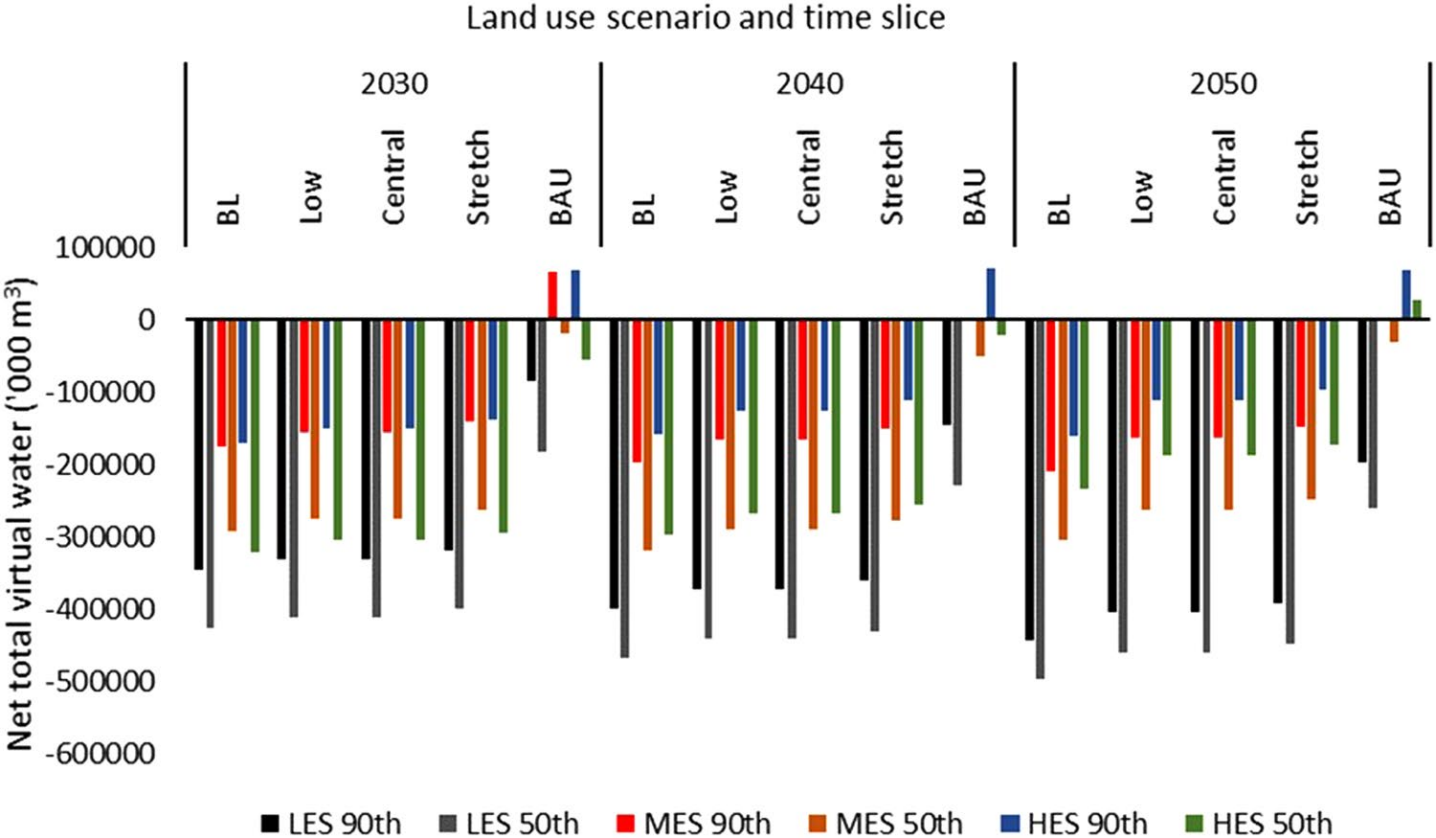
Net total virtual water flows (‘000 m^3^) based on malt barley balances.

Based on the malt barley balances, net virtual water flows (blue, green, and total) to the UK were estimated (Figs. 3–5). This virtual water flow is potential, assuming the UK would import malt barley to balance the observed deficits. Green virtual water (Fig. 3) accounted for 97% while blue virtual water (Fig. 4) accounted for 3% of total net virtual water flows to the UK (Fig. 5). For the most part, the UK would have net virtual water inflows. However, under the BAU, the UK could potentially export green virtual water (ranging from approximately 27,674 under the HES, 50^th^ percentile yield in the 2050s, to 70,984 thousand cubic metre under the HES, 90^th^ percentile yield in the 2040s) due to surplus malt barley supply under the MES and HES from the 2030s to the 2050s at the 90^th^ percentile yield, or the HES under the 50^th^ percentile yield in the 2050s (Fig. 3). As a result, the blue virtual water flows would be zero for these surpluses (Fig. 4) as the VWC of UK barley was 100% green. As would be expected, the net virtual water flows to the UK were lowest under the BAU and highest under the BL (Figs. 3–5).

The wider range of blue water inflows was 0–7.2 million cubic metre under the BAU, 155–484 million cubic metre for the BL, 123–448 million cubic metre for the Low and Central, and 2.7–12.4 million cubic metre under the Stretch scenario (Fig. 4). Similarly, excluding the pockets of surpluses that resulted in potential virtual water export under the BAU, the wider range of net total virtual water flows to the UK under the BAU was 20–261.6 million cubic metre (Fig. 5). The wider range was 159.5–498 million cubic metre for the BL, 111.9–460.7 million cubic metre for the Low and Central scenarios, and 97–449 million cubic metre for the Stretch scenario. Thus, the maximum net virtual water inflows under the BAU is within the range of the Stretch scenario but far below the ranges of the remaining land use scenarios. Thus, the patterns observed from the supply and deficit would hold for the virtual water flows.

## Discussion

The malt-based beverage industry, which plays important economic and sociocultural roles, subsists on stable supplies of malting barley. Yet, there is little information on the future supplies of malt barley and its implications for the sustainability of malt-based beverage industries and related downstream industries^4^. The current study adopted a food balance approach^10,19^ to estimate future gaps in demand and supply of malt barley in the UK under projected climate change, land use change due to land-based climate mitigation policies and aspirations, and population change. The food balance approach permits the estimate of shares of the utilization components of a given food commodity that has multiple end uses. Within the limits of the current study, the results show that the UK could face deficits in malt barley supplies in the future. The deficits under the BAU (which represents current land area under barley production) and at 90^th^ percentile yield ranged from 114 thousand tonnes under the LES in the 2030s to 259 thousand tonnes under the LES in the 2050s (Fig. 1). The corresponding deficits under the Stretch scenario (which represents the largest land area of barley and a focus on food production under the climate mitigation policy scenarios) ranged from 128 thousand tonnes under the HES to 517 thousand tonnes under the LES in the 2050s. The surpluses observed at some points under the BAU suggest that the observed deficits are largely due to reductions in land area allocated to barley production. In addition, this observation suggests that the effect of climate change alone, in combination with the current land area under barley production, could results in adequate malt barley supplies in the future as yields increased from the LES to the HES and from the 2030s to the 2050s, indicating the beneficial effects of elevated atmospheric carbon dioxide on barley grain yields^11^. However, based on the projected area of croplands^13^, the results suggest that climate mitigation policies can lead to large deficits in malt barley supply due to reductions in the land area of barley. This observation is consistent with that reported for feed barley supply in the UK^10^.

Total barley supply is contingent on domestic production and import. With production, yield and land area are the major determinants of total production while import is contingent on shortfalls in domestic production and availability on the international market. While yield is principally affected by genetic, environmental and management factors, land area allocated to barley will depend on policies and legislations, market signals, availability or profitability of alternatives or substitute crops, and ultimately farmers’ decisions on production mix. Spring barley yield in the UK is projected to remain resilient and potentially increase under projected climate change as faster accumulation of thermal time and higher atmospheric carbon concentration permit early maturity, avoidance of summer heat and water stresses, and higher yields^11^. This, together with the high quality of UK malt barley (and the exacting quality requirements of malt barley), could suggests that the UK would probably rely largely on domestic production to feed its malting industry (all things being equal). Hence, changes in land area allocated to barley would be a major determinant of total supply in future, and therefore the resilience of UK malting capacity in the future. Currently, cereals (mainly wheat and barley) accounts for over 50% of cultivated arable land area in the UK^7^. Evidence from several reports suggest that areas of croplands in the UK could reduce substantially in the future^14,15,27^ due mainly to policies related to climate change mitigation and ambitious targets on emission reductions in the UK^12,13,28,29^, with adverse consequences for barley production^10^. Using the Central and the Stretch scenarios, reductions in barley land area with regard to the BAU were 174.3 and 166.1 thousand ha in the 2030s, 157.9 and 149.8 thousand ha in the 2040s, and 141.6 and 133.4 thousand ha, respectively (data not shown). The deficits were largest under the BL scenarios which continues current and prevalent land-based climate mitigation activities (mainly biofuel production and afforestation). This suggests that while cropland area could increase gradually to the 2050s under climate change mitigation policies, the reductions in the near term are larger and overall reductions would be large enough to create deficits in supply relative to demand. It is probable that the UK would continue on this path of ambitious climate mitigation policies in response to its obligations and commitments under the UK Climate Change Act (2008), the EU Renewable Energy Directive (2009) and the United Nations Framework Convention on Climate Change (UNFCCC)^12,13^ and in the context of current pressures from civil societies and the Paris Agreement to reduce emissions. Barley is singled out amongst cereals to lose substantial area of land to biofuels in the EU^30^.

Globally, consumption of malt-based beverages is expected to increase in correspondence with anticipated rise in disposable incomes mainly in developing countries^2^. In developed countries, however, population growth will be the major driver of increase in demand for food and drink^31^, even in the face of health concerns^3^. It has been suggested that the impact of climate change on supply and access to ‘luxury goods’ could be larger than on staple foods^4^. Yet, this aspect of the impacts of climate change has received little attention. Given the economic and socio-cultural importance of the malting industry, and the sensitivity of the industry and its related downstream chains to instability in supply of malt barley, it is important to begin to give malt futures the appropriate level of research and policy attention. For example, it has been reported that extreme heat and drought events could decrease net global barley production, resulting in different degrees of impacts on beer prices and consumption in different countries^4^. Specifically, for the UK, the price of beer increased under all the four emission scenarios they used^4^. The EU accounts for over 60% of world malt trade and the UK has the second largest malt capacity in the EU^8^. In addition, the UK malting industry is the third largest in the world, supplying malt to fourteen of the twenty largest brewers worldwide^9^. These, together with the global prestige and premium value of UK whisky, show a need to take measures to address the projected deficits in UK malt barley supply.

From sustainability and policy perspective, it can be argued that a number of factors (beyond the farm scale) could affect total land area eventually allocated to spring or malt barley production. Principal amongst these would be market signals^10,15,32^. For example, low demand for malt-based beverages or products would lower the direct demand for malt barley, which in turn will affect farmers’ decisions regarding land allocation or production mix. In addition, increased profitability of other crops (e.g. biofuel crops) could result in shifting land to such crops. The sensitivities and sustainability of both the malt barley production and its dependent downstream industries (mainly the malting industry) are in a positive self-reinforcing feedback relationship. That is, a decrease in one result in a decrease in the other, and vice versa. The malting industry, however, is extremely sensitive to disruptions or instability in malt barley supply. Its sustainability is therefore contingent on stable production and supply of malt barley by farmers or by imports. The current study shows that while UK barley production would remain viable under rain-fed conditions, land use changes would be a principal determinant of supply from domestic production. This is important as, historically, agricultural land use change in the UK is known to be driven mainly by policy interventions, with other factors playing secondary roles^15,27^. Hence, it is likely that policies or legislations, especially regarding climate change and energy, could affect land area allocated to barley production, with direct consequences on the UK malting industry. Given that global barley production would likely shrink^4^, especially in the EU^30^ which is UK’s main barley trading partner, and with Brexit on the horizon, the malting industry would have to pay attention to domestic production. Imports could be expensive, unreliable and have quality issues that could render the UK malting industry less competitive. The scale of deficits observed in the current study, based on current level of consumption, gives cause for adaptive responses, foremost from policy level, aimed at securing and stabilizing future malt barley supplies if the malting industry is to remain sustainable and maintain its economic and socio-cultural roles in the UK and the world. To this end, policies that shrink the area of land for barley, and render malt barley production less attractive or competitive deserve careful analysis. The current study shows that even if current land area is maintained, the UK could have surpluses in malt barley supply by mid-century.

Import to balance the observed deficits would result in net virtual water flows to the UK (except where the surpluses were observed). The first challenge that needs to be addressed relates to Brexit as the EU is the main barley trading partner of the UK, and barley production is projected to decrease in the EU due to reductions in land area of barley^30^. The second challenge would be to assess and secure where else malt barley could be imported from as global barley production could decrease^4^. That notwithstanding, the UK could move from a net exporter of green water in barley grains to a net importer of blue virtual water. Total blue virtual water inflows could be as high as 448 million cubic metre for the Central scenario or 484 million cubic metre for the BL scenario (Fig. 4). This is in contrast to the maximum of 7.2 million cubic metre observed under the BAU. This shows that, at least, maintaining current land area under barley production could significantly lower future environmental footprint of the UK with regard to blue water inflows and global freshwater security. This blue water import would not be agri-compatible^33^, that is, it would not serve water-dependent import and so would amount to global freshwater loss. As a result, and with expected increase in global blue water scarcity, the UK could contribute to saving global freshwater by reducing its blue virtual water inflows associated with barley import, especially in countries that suffer or are projected to suffer water scarcity (e.g. Spain and Southern France). To our knowledge, the current study is the first attempt to estimate future balances and virtual water flows of malt barley at national scale using food balance approach and in the context of projected climate, land use and population change. A wider study would be appropriate to obtain a view of future malt barley balances and the direction of virtual water flows to support policy and trade decisions that limit impacts on freshwater resources and the sustainability of the UK and global malting industry.

While the limitations of the current study have been elaborated elsewhere^10,11^, some specific limitations are worth mentioning here. The adoption of a food balance approach implies that the proportionate distribution of barley to utilization components in the future would be the same as under the baseline. The proportionate distribution or allocation to malting could be altered mainly by changes in market conditions and malting quality. As explained earlier, decreased demand for malt-based beverages, for example, due to price or health concerns, would reduce demand for malt barley by the malting industry and vice versa. Higher profit margins for other utilization components of barley could also drive a shift in production (and for that matter supply) from malting to those other components. Similarly, higher profit margins for other crops (e.g. biofuels) could shift land away from barley to those other crops and thereby affect total supply of malt barley. While these issues are difficult to model and beyond the scope of the current study, they are noteworthy. Secondly, even though the quantity of malt barley supply is essential, changes in the malting quality of barley grains would be a major determinant of the final quantity of supply to the malting industry. Crucially, the grain nitrogen content is a major determinant of malting quality and this depends on soil fertility and other agronomic management practices such as sowing dates, and weather conditions. In the current study, the simulations were done under optimal fertility conditions (and so fertility stress was not an issue). It is recognized, however, that nitrogen content of barley grains is an important quality determinant for both feed and malt barley, and soil nitrogen dynamics can affect the yield gains from elevated atmospheric carbon dioxide. While this was beyond the scope of the current study, due to potential modelling complications, it is recognized that grain quality would remain a major determinant of the final quantity of supply to malting as a utilization component and the sustainability of the malting industry is, therefore, dependent on not just the quantity but also the quality of malt barley supply in the future. It is recommended that the combined effects of the dynamics of both soil water and nitrogen content on the malting quality of barley under projected climate change, though complicated, should be a target of future studies.

## Conclusions

Stable and sustainable malt barley supply is crucial for the sustainability of the malting industry both in the UK and globally. The malting industry is very sensitive and vulnerable to unstable malt barley supply. Within the limits of the current study, the UK could face deficits in malt barley supply. Given the position of the UK in the EU malting capacity and global supplies or trade, the observed deficits would have both national and international consequences. The observed deficits are principally due to reductions in cropland areas in response to climate mitigation and energy policies. It is interesting to note that even if the current land area is maintained, together with the medium or high emissions scenarios, and a realization of the 90^th^ percentile yield, the UK could continue to supply adequate quantities of malt barley and generate surplus at some point. Future demand for malt-based beverages would increase in both developing and developed countries. It is important for the UK, as a potential beneficiary of climate change with regard to barley yields, to maintain malting capacity to serve both domestic and international demands. This requires a re-assessment of land use policies or policies that directly or indirectly shrink croplands, especially those related to energy and climate change mitigation.

## Data Availability

Publicly available datasets were used. Other information or data used are available in the references within this paper.
